# Reproducible Non-Volatile Multi-State Storage and Emulation of Synaptic Plasticity Based on a Copper-Nanoparticle-Embedded HfO*_x_*/ZnO Bilayer with Ultralow-Switching Current and Ideal Data Retention

**DOI:** 10.3390/nano12213769

**Published:** 2022-10-26

**Authors:** Shuai Chen, Hao Chen, Yunfeng Lai

**Affiliations:** 1 Fujian Science & Technology Innovation Laboratory for Optoelectronic Information of China, Fuzhou 350108, China; 2 School of Physics and Information Engineering, Fuzhou University, Fuzhou 350108, China

**Keywords:** resistive switching, memristor, electronic synapse, oxides

## Abstract

The multilevel properties of a memristor are significant for applications in non-volatile multi-state storage and electronic synapses. However, the reproducibility and stability of the intermediate resistance states are still challenging. A stacked HfO*_x_*/ZnO bilayer embedded with copper nanoparticles was thus proposed to investigate its multilevel properties and to emulate synaptic plasticity. The proposed memristor operated at the microampere level, which was ascribed to the barrier at the HfO*_x_*/ZnO interface suppressing the operational current. Compared with the stacked HfO*_x_*/ZnO bilayer without nanoparticles, the proposed memristor had a larger ON/OFF resistance ratio (~330), smaller operational voltages (absolute value < 3.5 V) and improved cycle-to-cycle reproducibility. The proposed memristor also exhibited four reproducible non-volatile resistance states, which were stable and well retained for at least ~1 year at 85 °C (or ~10 years at 70 °C), while for the HfO*_x_*/ZnO bilayer without copper nanoparticles, the minimum retention time of its multiple resistance states was ~9 days at 85 °C (or ~67 days at 70 °C). Additionally, the proposed memristor was capable of implementing short-term and long-term synaptic plasticities.

## 1. Introduction

Memristors exhibiting multilevel resistive switching properties have attracted much attention from industries and academic communities due to their potential applications in non-volatile multi-state storage [1], reservoir computing [2], neuromorphic computing and in-memory computing [3,4]. The three-dimensional architecture of memristors has been considered as a hardware basis to fulfill the requirements of the aforementioned applications [5,6]. Embedded memristors might possess low power consumption and the so-called ON/OFF ratio of high-resistance states (HRS) to low resistance states (LRS) should be extended to accommodate as many storage states as possible. In addition, memristors should exhibit reproducible resistive switching behavior as a guarantee of reliable functionalities [7].

Several techniques have been attempted to control the dynamic evolution of resistance, which is helpful in achieving multi-state storage and simulating synaptic behavior [8]. Integrating multiple resistive switching mechanisms, such as ferroelectric [9], resistive and magnetic behaviors [10,11], into the same device seems to be an effective strategy. Through the modification of the device structure [12,13,14], the embedment of nanoparticles into the storage medium [15,16,17,18], the optimization of the electrical stimulus [19], element doping and interface modification [20,21], multi-state storage and synaptic plasticity could also be attained by precisely controlling the dynamic growth (or dissolution) of the conductive filaments (CFs) [22,23,24,25,26]. Stacked layers as a storage medium improve the stability and reliability of resistive switching to favor multi-state storage, but its lower ON/OFF resistance ratio is normally hard when accommodating many more intermediate resistance states. Doping metal nanoparticles into a storage medium facilitates the formation of CFs and usually extends the ON/OFF resistance ratio, but the relatively large operational current (~mA) does not serve its application in low-power neuromorphic computing [15,16,26,27]. Combining bilayers embedded with a nanostructure seems to be a feasible way to have a large ON/OFF resistance ratio as well as a low power consumption [28,29]. Niu Y combined an SiO_2_ limiting layer with MoS_2_ quantum dots (QDs) and inserted them into Al_2_O_3_/Pt. The SiO_2_ as a limiting layer provided a small fracture area in Al_2_O_3_ for reducing the power consumption. The MoS_2_ QDs positioned CFs to enhance the reproducibility. Sakellaropoulos D inserted Pt nanocrystals between the top electrode and the TiO_2−*x*_/TiO_2−*y*_ bilayer, in which the TiO_2−*y*_ layer with a lower oxygen content acted as series resistance to limit the current and the Pt nanocrystals also positioned CFs. However, the reproducibility and stability of intermediate resistance states are still challenging [19,26,30] since their retention at high temperature needs further improvements. New approaches are still worth investigation.

Hafnium oxide and zinc oxide are compatible with CMOS processing and exhibit excellent resistive switching properties [13,19,21]. Our previous work also indicates that the resistance of a HfO*_x_*/ZnO bilayer was closely associated with the barrier on the Hf*_x_*O/ZnO interface [31], which suggests the possibility of multilevel properties. Additionally, the HfO_x_ RRAM doped with Cu presented a multilevel non-volatile storage capability [32], which further provides another clue for multilevel properties. In order to combine an interface barrier to suppress the current and a nanostructure to position conductive pathways, we therefore designed a HfO*_x_*/ZnO double-layer embedded with copper nanoparticles and investigated its multi-state storage properties and conducted a simulation of its synaptic plasticity.

## 2. Materials and Methods

**Figure 1:**
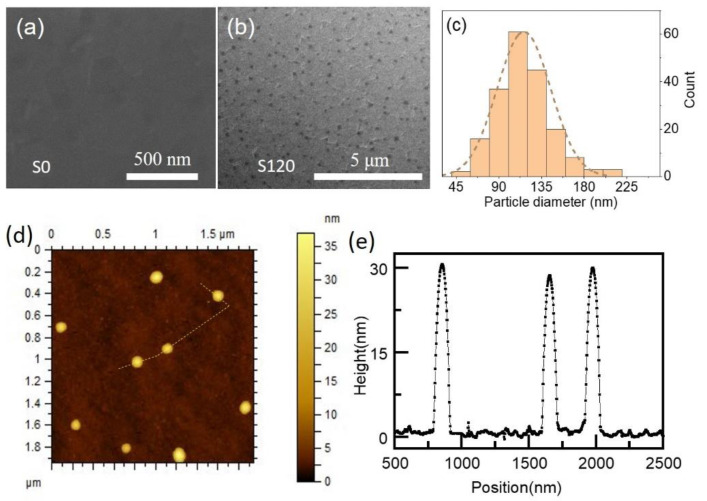
SEM images of HfO*_x_* thin films covered (**a**) without and (**b**) with copper nanoparticles; (**c**) diameter distribution of copper nanoparticles; (**d**) AFM image of the HfO*_x_* thin films covered by nanoparticles with (**e**) the surface profile along the yellow dotted line in (**c**).

**Figure 2:**
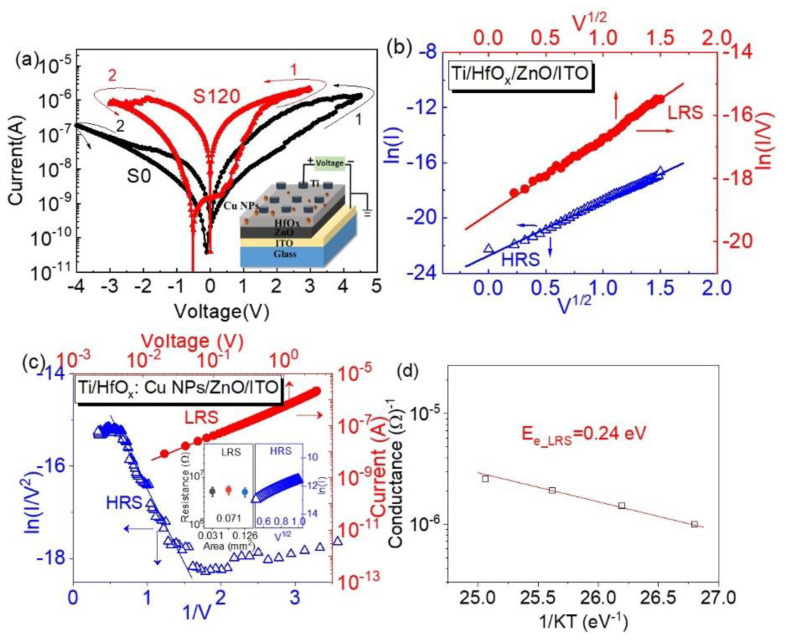
(**a**) Current–voltage characteristics of the stacked layers with the inset of schematic diagram of device structure and measurement configuration; (**b**) ln(*I*)-*V*^0.5^ and ln(*I*/*V*)-*V*^0.5^ fittings for the HRS and LRS of device S0; (**c**) ln(*I*/*V*^2^)-1/*V* and log(*I*)-log(*V*) fittings for the HRS and LRS of device S120. The right inset is the ln(*I*)-*V*^0.5^ fitting of the HRS at low electrical field, while the left inset is the LRS resistance vs. device area. (**d**) Conductance vs. 1/KT for the LRS device with Cu NPs.

A corner-covered ITO/glass substrate was used with ~15 nm sputter-deposited ZnO thin films on the surface to fabricate a HfO*_x_*/ZnO double-layer-based memristor. The ~15 nm HfO*_x_* thin films were then sputtered on the ZnO surface, followed by 10 s deposition of thin copper films. Subsequently, the samples were heated with rapid thermal processing at 550 °C for 20 s under a nitrogen atmosphere to form Cu nanoparticles. Finally, the upper ~15 nm HfO*_x_* thin films were deposited on the nanoparticles, followed by patterning a titanium electrode (Φ = 200 μm) with a shadow mask to complete the device fabrication. Devices without nanoparticles were also prepared for comparison and marked as S0. Preparation parameters were determined according to our previous works and reference [31,33]. The morphologies of the thermal-processed HfO*_x_* thin films were observed by scanning electron microscopy (SEM) and an atomic force microscope (AFM) as shown in Figure 1. Cu nanoparticles with an average diameter of ~120 nm (Figure 1c) originated from the 10-second-deposited Cu thin films and the corresponding devices were marked as S120. The surface densities of the Cu nanoparticles were ~2.88 × 10^8^ cm^−2^. Thermal processing drove the Cu thin film to cluster into ellipsoid-shaped nanoparticles with a height of ~30 nm as per the surface profile shown in Figure 1e. The switching properties of the devices were characterized using a semiconductor characterization system (4200-SCS; Keithley, Cleveland, OH, USA) with 0.5 V for reading. Electrical stimuli were applied to the top titanium electrodes with the ITO bottom electrodes grounded, as schematically shown in the inset of Figure 2a. To evaluate the capability of multi-state storage, the devices were set to be at several resistance states by controlling the amplitude of the electrical stimuli. The measurements were performed from room temperature to 200 °C to appraise the data retention and thermal stability of the storage states. The same semiconductor characterization system equipped with a pulse generator and oscilloscope was also used to study the synaptic behavior of the device with the bottom electrode as the post-synaptic location and the other as the pre-synaptic location.

## 3. Results and Discussion

### 3.1. Bistable Storage Properties

Figure 2a presents the current–voltage (*I*–*V*) characteristics of the devices. All the devices exhibited non-volatile bipolar resistive switching properties with operational currents less than 10 μA. The operational voltage decreased with the embedment of copper nanoparticles. To better understand the effects of the copper nanoparticles, the conduction mechanism of the devices was studied. Several physical models have been proposed to explain the conduction mechanisms of the devices. For the device S0 (Figure 2b), a good linear fitting of ln(*I*) versus *V*^0.5^ suggested that a Schottky emission mechanism dominated its HRS conduction, while its LRS conduction was governed by the Poole–Frenkel emission theory due to the linear fitting of ln(*I*/*V*) versus *V*^0.5^ [34,35]. The copper nanoparticles changed the conduction mechanisms of the devices (Figure 2c). For the HRS, the linear fitting of ln(*I*/*V*^2^)~1/*V* under a high electric field and the linear fitting of ln(*I*) versus *V*^0.5^ under a low electric field suggested that the HRS conduction of the device S120 changed from a Schottky-emission-dominated mechanism to a Fowler–Nordheim tunneling mechanism with the increase in the electric field. The LRS resistance did not show a clear trend with the device area (left inset of Figure 2c) and the linear log(*I*)~log(*V*) of its LRS indicated a filament-assisted Ohmic conduction [36]. We then applied the hopping model to the curve and plotted the dependence of the conductance vs. 1/KT, as shown in Figure 2d, to further obtain insights into the Ohmic conduction. The activation energy for electronic migration (E_e_) extracted from the curve was ~0.24 eV, which agreed with the literature reports and suggested it was a charge-trap device [37]. Non-metallic oxygen vacancies (V_o_s) might be closely associated with the conduction. However, considering the high diffusion coefficient of Cu, the role of metallic copper could not be totally excluded from the conduction.

**Figure 3:**
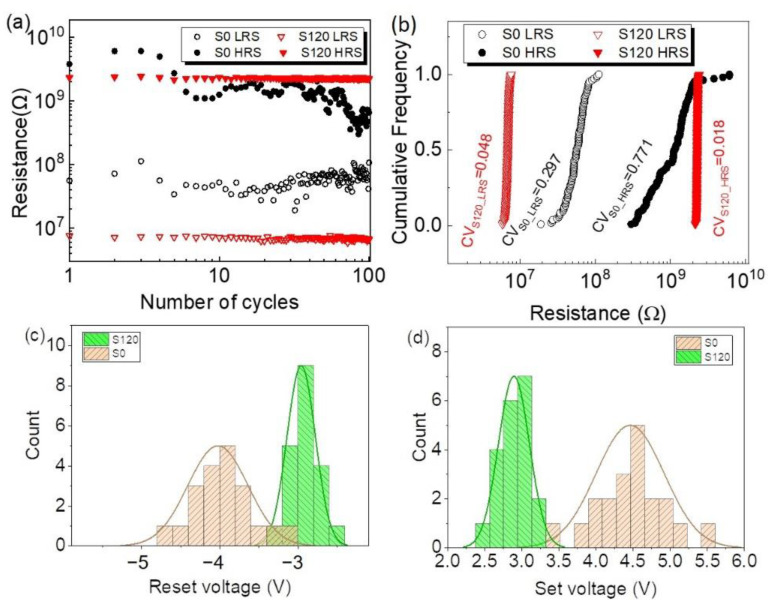
(**a**) The HRS and LRS resistances during 100 consecutive switching cycles with (**b**) their cumulative frequencies. Counts versus (**c**) reset voltage and (**d**) set voltage of devices.

To further investigate the non-volatile bistable storage properties, the resistances of the HRS and LRS during 100 consecutive switching cycles were studied. As presented in Figure 3a, the HRS resistances of the device S0 gradually decreased during the switching cycles, though its resistances were greater than those of device S120 at the beginning. As for the LRS, the resistances of device S120 were much smaller than those of device S0. As a result, the ON/OFF resistance ratio increased with the addition of the copper nanoparticles from ~20 to ~330 to provide a much larger space to accommodate multiple storage states. To evaluate the reproducibility of the device resistance, the coefficient of variation (CV), defined as the ratio of the standard deviation to the mean, was utilized. The resistance CVs for the HRS and LRS decreases with the addition of the copper nanoparticles, as shown in Figure 3b, suggesting an improved resistance reproducibility with the embedment of copper nanoparticles. To count the operational voltages, the set voltage was defined as the voltage setting the operational current at half of the maximum, while the reset voltage was defined as the voltage dropping the operational current to half of the maximum. We then randomly selected 20 devices from each device category to count the operational voltages. As shown in Figure 3c,d, most of the set voltages and the reset voltages decreased with the embedment of copper nanoparticles with the counts matching a Gaussian distribution (fitting lines in Figure 3c,d). The narrower full-width at half of the maximum (FWHM) for device S120 further suggested the improved uniformity with the addition of copper nanoparticles.

### 3.2. Multilevel Properties and Data Retention of Multiple Storage States

**Figure 4:**
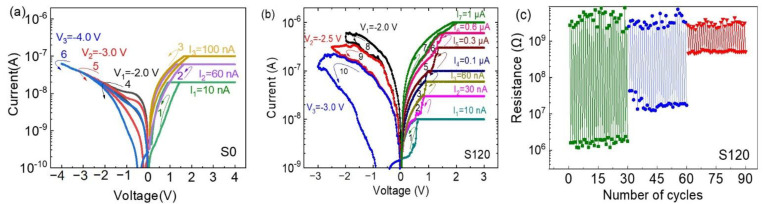
Multilevel properties of devices (**a**) without and (**b**) with copper nanoparticles; (**c**) Voltage-pulse-stimulated endurance cycling for 30 cycles per state of device S120.

The multilevel resistive switching properties of a memristor indicate several resistance states or conductance states that might be acquired by setting current-compliances (*I*_c_) or voltage-compliances (*V*_c_). If the conductance states represent synaptic weights, the simulations of synaptic behaviors by the device might be possible. If the resistance states are sustainable, non-volatile multi-state storage might be realized. Therefore, the multilevel resistive switching properties of the devices were then evaluated by setting gradually increasing values of *I*_c_. As shown in Figure 4a, the device S0 completed its set process by three rounds with three increased values of *I*_c_. We observed that the beginning of the *I*–*V* curve of each round did not overlap the end of the *I*–*V* curve of the last round, which indicated that the ending resistance of the last round was probably not stable. Three rounds were also required to complete the reset process with three increased values of *V*_c_. Similarly, the *I*–*V* curves of the successive rounds did not overlap during the reset process, suggesting the possibly instable resistance of the storage states. However, the embedment of copper nanoparticles brought some changes. The beginning of the *I*–*V* curve of each round well overlapped the end of the *I*–*V* curve of the last round during the set process and the reset process as shown in Figure 4b, which indicated that the ending resistance of each round might be reserved to favor multi-state storage. The easy identification of storage states is another important factor for multi-state storage, but the ON/OFF resistance ratio of device S0 was much smaller than that of device S120. The identification of multiple storage states in device S0 might not be easy as instable resistance states challenge the effective identification of states with a small ON/OFF resistance ratio (~20), so device S120 might thus be a better candidate for multistate storage. Therefore, voltage stimuli were applied to device S120 in an initial HRS. Reproducible resistance switching to intermediate resistance states was observed in Figure 4c with acceptable resistance fluctuations, which allowed us to identify four repeatable storage states.

**Figure 5:**
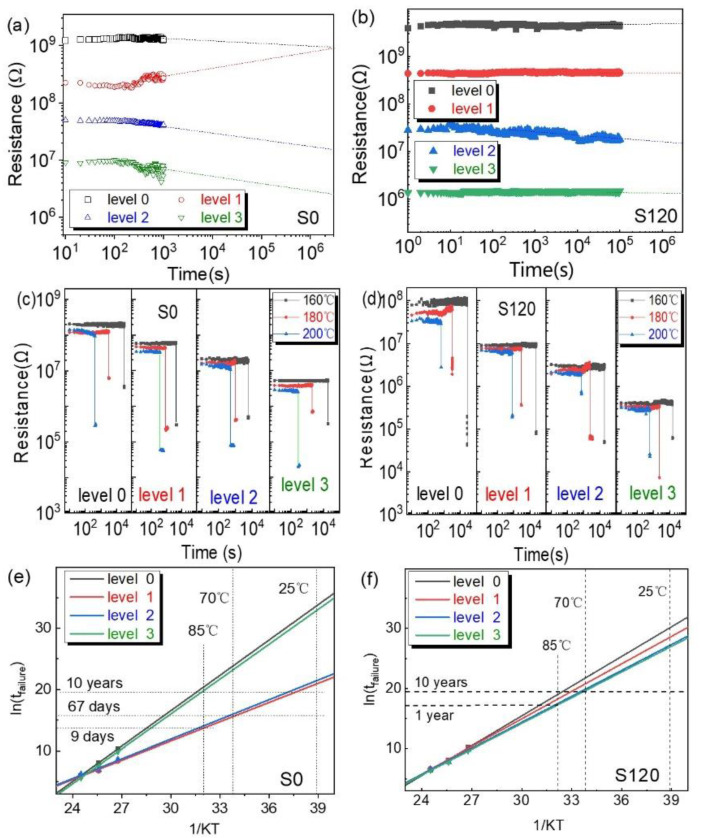
Room temperature data retention of four storage states for (**a**) device S0 and (**b**) S120; dependence of resistance on time at high temperatures for (**c**) devices S0 and (**d**) S120; temperature dependence of retention-failure-time fitting lines following the Arrhenius equations for (**e**) devices S0 and (**f**) S120.

Data retention is significant for practical applications in non-volatile multistate storage. Both devices were set to four resistance states, and their resistance tracking at room temperature is shown in Figure 5a,b. All storage states of devices S0 and S120 could be well distinguished during measurement. The resistance of device S0 fluctuated more than that of device S120, and its level 0 and 1 could not be identified after 10^6^ s. To further appraise the sustainability of each resistance state, the accelerated retention test for resistance states under various temperatures was performed, which has been widely accepted to characterize the retention properties of RRAMs in a time-efficient manner. In this test, the retention failure time (*t*_failure_) is measured as a function of temperature at elevated temperatures and then extrapolated to lower, nominal operating temperatures [38,39,40]. For the oxide-based RRAM in this work, the resistance evolution was closely associated with the migration of defects. Therefore, the retention failure process was dominated by Fick diffusion [40,41], which has an Arrhenius temperature dependence, i.e., *D* ∝ exp(−*E*_a_/k_B_*T*), where *D* is the diffusivity, *E*_a_ is the Fick diffusion activation energy for the migration of defects (e.g., V_o_s and Cu ions in this work), k_B_ is the Boltzmann constant and *T* is the temperature. As a result, the *t*_failure_ is inversely proportional to *D* and can be expressed as Arrhenius equation *t*_failure_ ∝ exp(*E*_a_/k_B_*T*) [39,42,43]. The *t*_failure_ at lower temperatures could be achieved using linear extrapolation from the data measured at high temperatures. In this work, the retention failure time was defined as the time for the abrupt change in resistance as shown in Figure 5c,d. Table 1 lists the retention failure times of resistance states under different temperatures, according to which the embedment of Cu nanoparticles extended the retention failure times of intermediate resistance states at higher temperatures. As shown in Figure 5e,f, we then plotted the mean retention failure time vs. the reciprocal temperature to conclude the data-retention abilities of all the resistance states by using the Arrhenius equation. The retention times of the resistance states at lower temperatures were thus available by the extraction results of the Arrhenius equation. All the resistance states of device S120 could be well kept for >10 years at 70 °C to satisfy practical applications. The HRS (level 0) and LRS (level 3) of device S0 could be kept for more than 600 years at 70 °C, but its intermediate resistance states (level 1 and level 2) could merely be retained for 67 days at 70 °C (or 9 days at 85 °C), which impedes its practical applications. To further analyze the effects of the nanoparticles for data retention, the value of *E*_a_s of the storage states was calculated and is listed in Table 2. According to the literature [44,45], the activation energy of the oxygen vacancies in ZnO is ~1.8 eV. The activation energy of the oxygen vacancies in HfO_x_ is 0.4–2.13 eV, which is closely associated with the composition, density and crystal structure of the material. The lower density inside the CFs could increase the confinement of the oxygen ion diffusion in the CF region and stabilize the CFs. The HRS (level 0) and LRS (level 3) of device S0 had much higher activation energies (1.92 eV and 1.90 eV) to stabilize defect status and sustain a rather long retention time. However, its intermediate resistance states (level 1 and level 2) had much lower activation energies (~1 eV). Compared with the HRS and LRS, the migration of defects for the intermediate states might destroy the stability of conductive pathways, exhibiting poor retention. Device S120 was on the contrary; the activation energies of the HRS and LRS decreased to 1.65 eV and 1.43 eV, respectively. However, the activation energies of the intermediate states increased through the embedment of copper nanoparticles, which balanced the retention of all the resistance states to the meet basic requirements of practical applications.

All the above improvements in non-volatile multi-state storage were attributed to the embedded copper nanoparticles and the stacked HfO*_x_*/ZnO layers. A Schottky barrier at the HfO*_x_*/ZnO interface suppressed operational current. Poole–Frankel conduction in the LRS of device S0 resulted in states with a much higher resistance. The embedded Cu nanoparticles significantly affected the whole process due to the electric field enhancement around the metallic nanoparticles [46]. As the embedded nanoparticles changed the LRS conduction mechanism to be CF-assisted Ohmic conduction with a much lower resistance, the ON/OFF resistance ratio of the device S120 was indeed broadened and possibly accommodated an increasing number of distinguishable resistance states. Meanwhile, the enhanced electric field surrounding the nanoparticle positions the CFs in the storage medium and secures the reproducibility of the resistance states, resulting in the reliable functionalities of multi-state storage. Furthermore, the activation energy reflected the difficulty in the migration of defects and was closely associated with the stability and retention of the resistance states. The embedded Cu nanoparticles serving as relays to construct conductive pathways shortened the spacing to build conductive pathways across the storage medium. The retention of the HRS (level 0) and LRS (level 3) degraded with the decreased values of *E*_a_s of 1.65 eV and 1.43 eV, respectively. However, for the intermediate resistance states (level 1 and level 2), an increasing number of defects (e.g., V_o_s and Cu ions) produced by the enhanced electric fields prevented the conductive pathways from being destroyed by the migration of a small number of defects. Considering the stacked HfO*_x_*/ZnO structure favoring resistance stability at a high temperature [47], the localized conductive pathways should thus be strengthened with increased values of *E*_a_s. The stability of the intermediate resistance states should be ensured as well to extend their retention. Consequently, reproducible non-volatile multi-state storage was obtained with desirable data retention. To highlight the merits of the HfO*_x_*/ZnO bilayer embedded with Cu nanoparticles, we compared the multilevel properties in this work with the other literature reports as listed in Table 3. It is obvious that the proposed HfO*_x_*/ZnO bilayer embedded with Cu nanoparticles had two advantages over the other reports. Firstly, four resistance states could be well reserved for ~1 year at 85 °C (or ~10 years at 70 °C) compared with the maximal retention of <10^5^ s in the other work. Secondly, the maximal operational current in this work was 10 μA, which was far smaller than that in the other work.

We should also note that the performance of a memristor is closely associated with the thickness of thin films that might change the conduction mechanism and affect performance. ZnO thin films with a suitable thickness strengthen the barrier on the HfO*_x_*/ZnO interface with an ignorable resistance contribution [31]. If the ZnO thin films are very thin or even disappear, the ZnO might not be able to feed the HfO*_x_* films with enough oxygen ions. The HfO*_x_* films would be in a less-insulative state, and the switching current would increase with a degraded barrier. Additionally, the prototype device in this work provided a cue for non-volatile multi-state storage. However, the diameter of the devices was ~200 μm, which was huge. Devices of a smaller diameter and with smaller Cu nanoparticles might be necessary for practical integration. Furthermore, though the experimental data presented that device S120 had satisfactory electrical properties and a data retention of ~1 year at 85 °C for four states, the potential evolution of the electrical properties might be discussed as well because the migration of elements in the bilayer structure might have effects on the electrical properties. The diffusion of hafnium and zinc atoms would degrade or even eliminate the HfO*_x_*/ZnO interface, causing the operational current to probably increase. Copper diffusion eventually leads to a Cu-doped storage medium, the multilevel properties might be reserved, and the device would have a large operational current [32]. Further investigations should be carried out to gain insight into the above issue.

### 3.3. Simulation of Synaptic Behaviors

**Figure 6:**
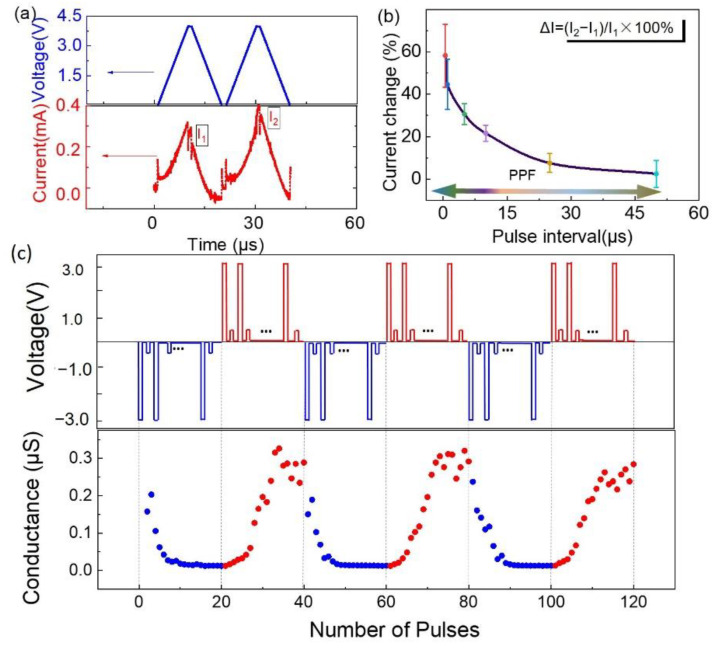
Paired-pulse facilitation (PPF) implementations of the NP-embedded devices with tunable conductance to emulate potentiation (red) and depression (blue); (**a**) excitatory postsynaptic currents (EPSC) in red with their amplitudes of I_1_ and I_2_ stimulated by a pair of presynaptic voltage spikes (blue); (**b**) current change, defined as (I_2_ − I_1_)/I_1_ × 100%, plotted as a function of pulse interval times; (**c**) gradual conductance modulation for the devices under pulse stimulation is shown on the top.

The multilevel resistive switching properties of the devices favored the simulation of synaptic plasticity to serve neuromorphic computing. Considering the improved multilevel resistive switching properties caused by the embedment of copper nanoparticles, we therefore studied the synaptic plasticity of device S120 to evaluate its potential to be an electronic synapse. Typical excitatory postsynaptic currents (EPSC) are presented with electrical stimulation in Figure 6a. The second EPSC was much higher than the first one in mimicking paired-pulse facilitation (PPF) in a bio-synapse. According to the above discussion, the trapped charges affected the resistance of the memristor. When the second electrical stimulation was close to the first one, the trapped charges triggered by the first stimulation did not have enough time to escape completely. Eventually, the shorter interval time of the stimulation was, the higher the intensity of EPSC obtained, as shown in Figure 6b. As a significant property of electronic synapses, the tunable electrical conductance of the devices was studied and triggered by electrical stimuli, as schematically shown in the upper side of Figure 6c. A voltage of 0.5 V was applied for reading the conductance after each potentiation and depression stimulus. The tunable electrical conductance upon potentiation and depression pulses is shown in Figure 6c, suggesting the capability of implementing synaptic weight modulation in response to a potentiation or depression stimulus.

**Figure 7:**
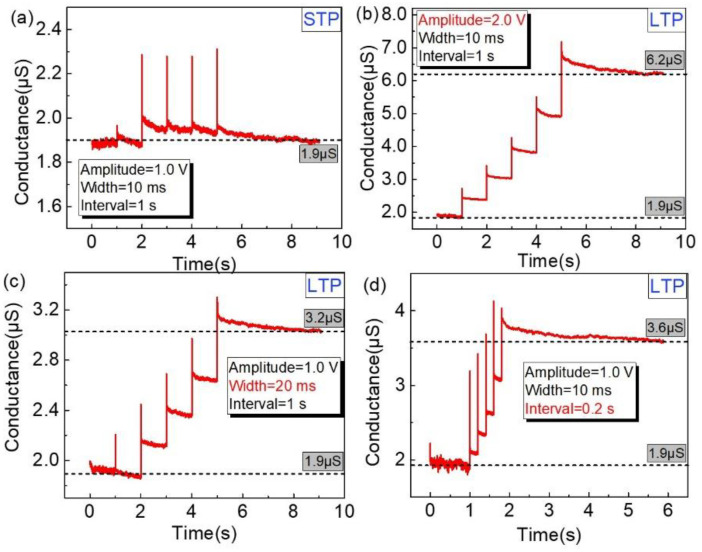
Implementation of transformation from STP to LTP for the NP-embedded devices with the insets of stimuli parameters; the transformation from (**a**) STP to LTP was realized through (**b**) increasing the stimuli amplitude, (**c**) extending the stimuli width, or (**d**) enlarging interval times between stimuli.

Short-term plasticity (STP) and long-term plasticity (LTP) are fundamental to memory in the human brain [48,49]. Electrical pulses with an amplitude (*V*) of 1.0 V or 2.0 V, a width (*W*) of 10 ms or 20 ms and repetition intervals (*T*) of 0.2 s or 1.0 s were applied to the memristors, as marked in the insets of Figure 7, to evaluate the capability of simulating synaptic plasticity. When input pulses were applied with the lower amplitude of 1.0 V, longer intervals of 1 s and a smaller width of 10 ms, the memristor failed to maintain the higher conductance state (~2.3 µS) and decreased with time back to its initial low conductance value, as shown in Figure 7a, suggesting behavior similar to the STP in a biological synapse. The memristor also presented a long-lived transition to the higher-conductance state with a higher amplitude of 2.0 V, a larger width of 20 ms or shorter intervals of 0.2 s as shown in Figure 7b–d, respectively, which implemented the LTP of a biological synapse as a permanent transition to higher-conductance states in response to the repeated application of input pulses.

**Figure 8:**
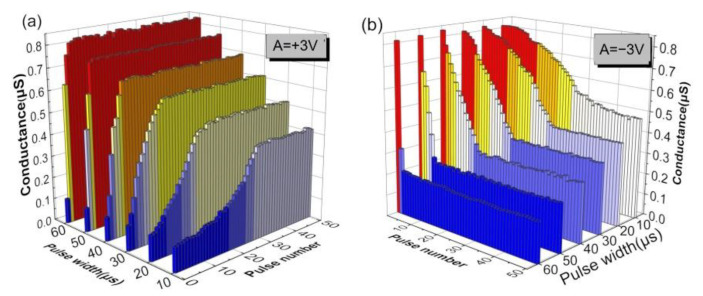
Conductance tunability upon pulse width during (**a**) potentiation and (**b**) depression for the NP-embedded devices.

**Figure 9:**
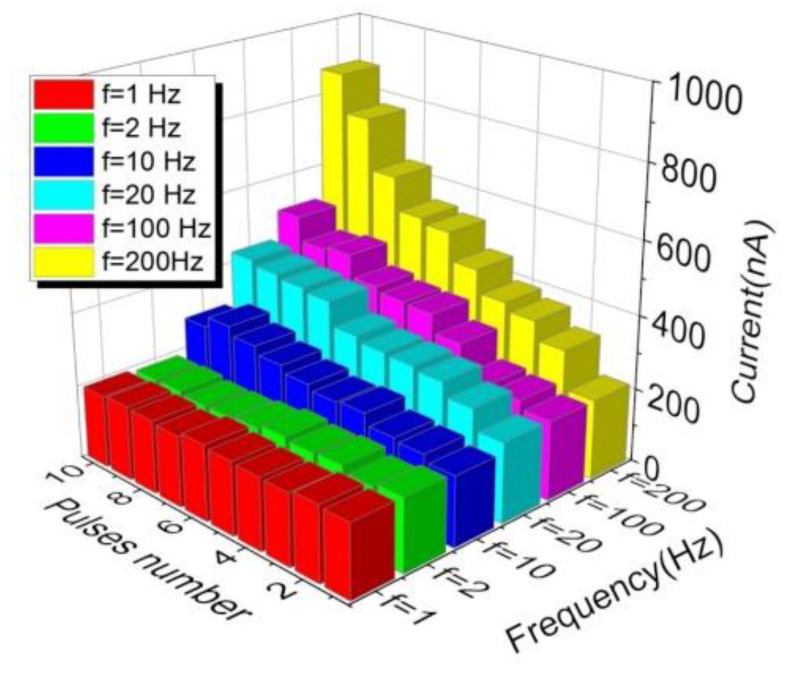
Current tunability upon stimuli frequency for the NP-embedded devices.

The synaptic weight in response to electrical stimuli was worth investigating as well. Figure 8 shows the dependence of conductance on the pulse width and pulse number, with the pulse amplitude and interval fixed at ±3 V and 1 ms, respectively. A larger pulse width induced a steeper change in conductance, showing a more digital resistive switching feature. In comparison, a smaller pulse width usually leads to a slow shift in conductance, presenting a more analog switching behavior. When an increasing number of electrical pulses is applied to a memristor, the conductance gradually increases in the potentiation process and decreases in the depression process, approaching an eventually stable conductance state to avoid the uncontrolled growth of synaptic strength and excessive neural firing [49]. Additionally, the pulse frequency affects the conductance of the memristor as well. Figure 9 shows the dependence of current on the pulse number and frequency, with the pulse amplitude and width fixed at 1.5 V and 50 µs, respectively. Instead of the ignorable current change caused by lower frequency pulses, higher frequency (10–200 Hz) pulses resulted in a much larger current variation with an increasing number of pulses. Therefore, the conductance variation in response to pulse was determined by the parameters of the applied pulses. Stimuli with a high energy flux triggered a larger change in conductance.

**Figure 10:**
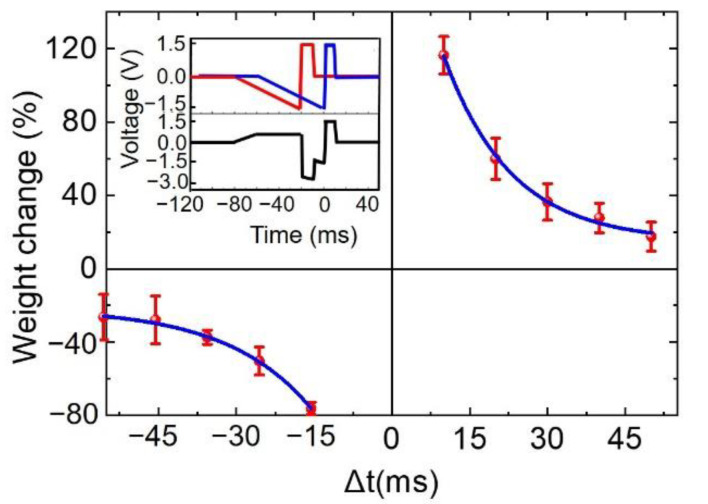
Implementation of the STDP learning rule for the NP-embedded devices. The synaptic weight change (Δ*w*) was tested as a function of the temporal difference (Δ*t*) between the pre-spike (blue) and post-spike (red) that are schematically shown in the inset. Equivalent stimulus in black applied to the device is schematically shown in the inset with Δ*t* = 20 ms. Δ*w* is defined as (*w*_2_ − *w*_1_)/*w*_1_, where *w*_1_ and *w*_2_, respectively, represent synaptic weights before and after stimulus.

As one of the necessary activity-dependent plasticities required to implement important synaptic learning rules for neuromorphic computing [50,51], spike-timing-dependent plasticity (STDP) has been widely investigated for an asymmetric form of Hebbian learning induced by the tight temporal correlation between the pre- and post-synaptic spikes applied to synaptic neurons, as shown in Figure 10. STDP indicates the change in synaptic efficacy determined by the timing of the activity of pre- and post-synaptic neurons, which means the synaptic weight change (Δ*w*) is a function of the temporal difference (Δ*t*) between the pre- and post-synaptic spikes. Schematic diagrams of the pre- and post-synaptic spikes with the equivalent stimulus to the device are presented in the inset of Figure 10. As shown in Figure 10, the synaptic weight was enhanced when the pre-synaptic spike led to the postsynaptic spike (Δ*t* > 0) with the occurrence of long-term potentiation, but it weakened when realizing long-term depression if Δ*t* < 0. A larger |Δ*t*| produced a smaller Δ*w*, suggesting a good agreement with the asymmetric Hebbian learning rule. The measurements were performed five times for statistical purposes. Therefore, the STDP learning rule was implemented in the memristor. Small cycle-to-cycle variations in Δ*w* were another notable feature, which might be attributed to the embedded NPs sustaining the reproducibility and reliability of the resistive switching process.

The embedment of copper nanoparticles into the storage medium benefitted the simulation of synaptic plasticity. The copper nanoparticles acted as relays of conductive pathways and stabilized them to ensure the reproducibility of the synaptic weight changes in response to an electrical stimulus. Additionally, the copper nanoparticles enhanced the device sensitivity to electrical stimuli and the device responded to stimuli with a smaller step-size. For one thing, the copper nanoparticles produced many more intermediate states to symbolize the synaptic weight and favor higher accuracy neuromorphic computing to implement the STDP learning rule. For another, electrical stimuli with low energy flux could produce unsustainable conductive pathways for mimicking the STP and the stimuli with high energy flux resulted in LTP due to the sustainable conductive paths. The transformation from the STP to LTP was also mimicked by controlling the energy flux of stimuli.

## 4. Conclusions

HfO*_x_*/ZnO bi-layer-based memristors with embedded copper nanoparticles were investigated. The embedment of Cu nanoparticles maintained the HRS conduction governed by a Schottky emission mechanism at a low electric field but changed the HRS conduction to cause it to be driven by a Fowler–Nordheim tunneling mechanism under a high electric field. The embedded Cu nanoparticles also changed the LRS conduction dominated by the Poole–Frenkel emission mechanism to be an Ohmic behavior associated with defect-assisted CFs. As a result, the copper nanoparticles acting as the relays of conductive pathways enhanced the localized electric fields to increase the stability and reproducibility of intermediate resistance states. The memristors thus operated at microampere levels with at least four reproducible non-volatile storage states, which could be retained for >10 years at 70 °C. Additionally, the copper-nanoparticle-embedded memristor was capable of implementing the short-term plasticity and long-term plasticity of a bio-synapse. The stacked HfO*_x_*/ZnO bi-layer embedded with copper nanoparticles opens up a route to reproducible non-volatile multi-state storage as well as electronic synapses.

## Figures and Tables

**Table 1:** Retention failure times of resistance states.

States	Device S0 (s)			Device S120 (s)		
	@160 °C	@180 °C	@200 °C	@160 °C	@180 °C	@200 °C
level 0	31,370	3280	440	26,340	3260	650
level 1	4770	990	450	25,170	3000	820
level 2	5720	1000	620	19,320	3380	750
level 3	20,200	2400	310	15,240	2400	620

**Table 2:** Activation energies of storage states for devices S0 and S120.

Device	Level 0 (eV)	Level 1 (eV)	Level 2 (eV)	Level 3 (eV)
S0	1.92	1.05	1.07	1.90
S120	1.65	1.53	1.44	1.43

**Table 3:** Comparison of multilevel properties.

Device Structure	R_OFF_/R_ON_	Retention (s)	Maximal Current (mA)	Reference
Ag/Ga_2_O_3_:PbS QDs/Pt	10^6^	10^4^ at 85 °C for three states	100	[18]
TaN/HfO_2_/Al_2_O_3_/HfO_2_/ITO	10^2^	10^4^	1	[13]
Al/PMMA/ZnO QDs/PMMA/ZnO QDs/PMMA/FTO	10^2^	5 × 10^3^	20	[15]
TaN/CeO_2_/Ti (1 nm)/CeO_2_/Pt	>10^2^	10^4^ at 85 °C	10	[16]
Ag/IGZO/MnO/Pt	10^6^	5 × 10^4^ at 80 °C	5	[22]
Al/AlOy/SnO_x_/FTO	20	5 × 10^3^	20	[25]
ITO/HfAlO/Pt NPs/HfAlO/ITO	>10	10^4^	3	[27]
Cu/AlO_x_/Al_2_O_3_/Pt	10^5^	10^4^	10	[30]
Ti/HfO_x_/Cu NPs/HfO_x_/ZnO/ITO	~3 × 10^2^	~1 year at 85 °C for 4 states	<0.01	This work

## Data Availability

Data can be available upon request from the authors.
